# Bis{1-[3-(diethyl­ammonio)­propyl­imino­meth­yl]naphthalen-2-olato}nickel(II) dinitrate

**DOI:** 10.1107/S1600536810025663

**Published:** 2010-07-03

**Authors:** Xiao-Hui Ji, Jiu-Fu Lu

**Affiliations:** a School of Chemistry and Environmental Science, Shaanxi University of Technology, Hanzhong 723000, People’s Republic of China

## Abstract

The asymmetric unit of the title compound, [Ni(C_18_H_24_N_2_O)_2_](NO_3_)_2_, consists of one half of the centrosymmetric nickel(II) complex cation and a nitrate anion. The Ni^II^ atom, lying on an inversion center, is four-coordinated by the phenolate O atoms and imine N atoms of two Schiff base ligands, forming a square-planar geometry. The O- and N-donor atoms are mutually *trans*. In the crystal structure, the nitrate anions are linked to the complex cations by inter­molecular N—H⋯O hydrogen bonds.

## Related literature

For background to complexes with Schiff bases, see: Hamaker *et al.* (2010 ▶); Wang *et al.* (2010 ▶); Mirkhani *et al.* (2010 ▶); Liu & Yang (2009 ▶); Keypour *et al.* (2009 ▶); Adhikary *et al.* (2009 ▶); Peng *et al.* (2009 ▶). For similar nickel complexes, see: Bhatia *et al.* (1983 ▶); Kamenar *et al.* (1990 ▶); Connor *et al.* (2003 ▶); Lacroix *et al.* (2004 ▶).
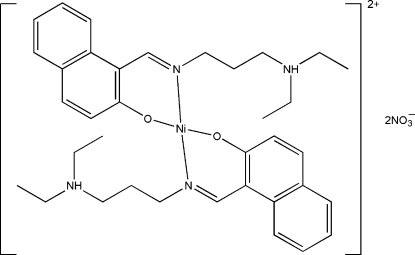


## Experimental

### 

#### Crystal data

[Ni(C_18_H_24_N_2_O)_2_](NO_3_)_2_
*M* *_r_* = 751.51
Monoclinic, 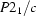

*a* = 11.096 (2) Å
*b* = 12.773 (3) Å
*c* = 12.743 (3) Å
β = 107.66 (3)°
*V* = 1720.9 (6) Å^3^
*Z* = 2
Mo *K*α radiation
μ = 0.63 mm^−1^
*T* = 298 K
0.22 × 0.20 × 0.20 mm

#### Data collection

Bruker APEXII CCD area-detector diffractometer
Absorption correction: multi-scan (*SADABS*; Sheldrick, 2004 ▶) *T*_min_ = 0.875, *T*_max_ = 0.885
14203 measured reflections
3689 independent reflections
2616 reflections with *I* > 2σ(*I*)
*R*_int_ = 0.055

#### Refinement

*R*[*F* ^2^ > 2σ(*F* ^2^)] = 0.069
*wR*(*F* ^2^) = 0.209
*S* = 1.05
3689 reflections
237 parameters
10 restraints
H atoms treated by a mixture of independent and constrained refinement
Δρ_max_ = 1.12 e Å^−3^
Δρ_min_ = −0.56 e Å^−3^

### 

Data collection: *APEX2* (Bruker, 2004 ▶); cell refinement: *SAINT* (Bruker, 2004 ▶); data reduction: *SAINT*; program(s) used to solve structure: *SHELXS97* (Sheldrick, 2008 ▶); program(s) used to refine structure: *SHELXL97* (Sheldrick, 2008 ▶); molecular graphics: *SHELXTL* (Sheldrick, 2008 ▶); software used to prepare material for publication: *SHELXTL*.

## Supplementary Material

Crystal structure: contains datablocks global, I. DOI: 10.1107/S1600536810025663/sj5031sup1.cif

Structure factors: contains datablocks I. DOI: 10.1107/S1600536810025663/sj5031Isup2.hkl

Additional supplementary materials:  crystallographic information; 3D view; checkCIF report

## Figures and Tables

**Table:** 

Ni1—N1	1.743 (3)
Ni1—O1	1.888 (3)

**Table:** 

N1^i^—Ni1—N1	180.0 (3)
N1^i^—Ni1—O1	88.51 (14)
N1—Ni1—O1	91.49 (14)

Symmetry code: (i) 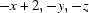.

**Table 2:** Hydrogen-bond geometry (Å, °)

*D*—H⋯*A*	*D*—H	H⋯*A*	*D*⋯*A*	*D*—H⋯*A*
N2—H2⋯O3^ii^	0.89 (6)	2.05 (4)	2.836 (8)	146 (6)
N2—H2⋯O2^ii^	0.89 (6)	2.17 (5)	3.033 (9)	162 (6)

Symmetry code: (ii) 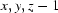.

**Fig. 1.**
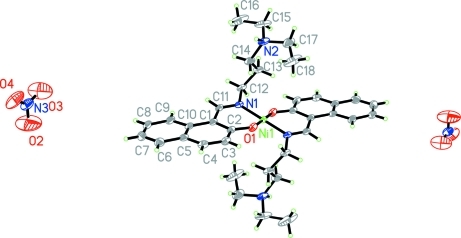
The molecular structure of the title complex, showing 30% probability displacement ellipsoids and the atom-numbering scheme. Unlabeled atoms are related to labeled atoms by the symmetry operation 2 - x, - y, - z.

**Fig. 2.**
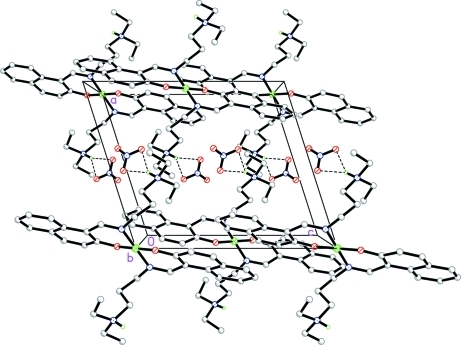
Crystal packing of the title compound with hydrogen bonds drawn as dashed lines.

## supplementary crystallographic information

### Comment

Schiff bases are known to be versatile ligands in coordination chemistry (Hamaker *et al.*, 2010; Wang *et al.*, 2010; Mirkhani *et al.*, 2010; Liu & Yang, 2009). A large number of complexes with Schiff bases have been reported because of their interesting structures and potential applications (Keypour *et al.*, 2009; Adhikary *et al.*, 2009; Peng *et al.*, 2009). We report here the crystal structure of the title new nickel complex with the Schiff base ligand 1-[(3-diethylaminopropylimino)methyl]naphthalen-2-ol.

The compound consists of a centrosymmetric mononuclear nickel complex cation and two nitrate anions (Fig. 1). The Ni atom, lying on the inversion center, is four-coordinated by two phenolate O atoms and two imine N atoms from two Schiff base ligands, forming a square planar geometry. The bond lengths (Table 1) around the Ni atom are comparable to those observed in similar nickel complexes (Bhatia *et al.*, 1983; Kamenar *et al.*, 1990; Connor *et al.*, 2003; Lacroix *et al.*, 2004).

In the crystal structure, the nitrate anions are linked to the complex cations by intermolecular N2—H2···O2 and N2—H2···O3 hydrogen bonds.

### Experimental

2-Hydroxy-1-naphthaldehyde (0.1 mmol, 17.2 mg) and *N,N*-diethylpropane-1,3-diamine (0.1 mmol, 13.0 mg) were mixed and stirred in methanol (10 ml) for 30 min. Then a methanol solution (5 ml) of nickel nitrate (0.1 mmol, 29.1 mg) was added to the mixture. The final mixture was stirred for another 30 min to give a red solution. Single crystals suitable for X-ray diffraction were obtained by slow evaporation of the solution at room temperature.

### Refinement

H2 atom was located from a difference Fourier map and refined isotropically, with the N–H distance restrained to 0.90 (1) Å, and with U_iso_(H) fixed at 0.08 Å^2^. The remaining H atoms were positioned geometrically (C—H = 0.93–0.97 Å) and refined using a riding model, with with *U*_iso_(H) = 1.2*U*_eq_(C) and 1.5*U*_eq_(C_metyl_). Rotating group models were used for the methyl groups.

### Crystal data

**Table:**

[Ni(C_18_H_24_N_2_O)_2_](NO_3_)_2_	*F*(000) = 796
*M**_r_* = 751.51	*D*_x_ = 1.450 Mg m^−^^3^
Monoclinic, *P*2_1_/*c*	Mo *K*α radiation, λ = 0.71073 Å
Hall symbol: -P 2ybc	Cell parameters from 1931 reflections
*a* = 11.096 (2) Å	θ = 2.3–24.5°
*b* = 12.773 (3) Å	µ = 0.63 mm^−^^1^
*c* = 12.743 (3) Å	*T* = 298 K
β = 107.66 (3)°	Block, red
*V* = 1720.9 (6) Å^3^	0.22 × 0.20 × 0.20 mm
*Z* = 2	

### Data collection

**Table:**

Bruker APEXII CCD area-detector diffractometer	3689 independent reflections
Radiation source: fine-focus sealed tube	2616 reflections with *I* > 2σ(*I*)
graphite	*R*_int_ = 0.055
ω scans	θ_max_ = 27.0°, θ_min_ = 1.9°
Absorption correction: multi-scan (*SADABS*; Sheldrick, 2004)	*h* = −14→14
*T*_min_ = 0.875, *T*_max_ = 0.885	*k* = −16→16
14203 measured reflections	*l* = −15→15

### Refinement

**Table:**

Refinement on *F*^2^	Primary atom site location: structure-invariant direct methods
Least-squares matrix: full	Secondary atom site location: difference Fourier map
*R*[*F*^2^ > 2σ(*F*^2^)] = 0.069	Hydrogen site location: inferred from neighbouring sites
*wR*(*F*^2^) = 0.209	H atoms treated by a mixture of independent and constrained refinement
*S* = 1.05	*w* = 1/[σ^2^(*F*_o_^2^) + (0.1045*P*)^2^ + 2.3396*P*] where *P* = (*F*_o_^2^ + 2*F*_c_^2^)/3
3689 reflections	(Δ/σ)_max_ < 0.001
237 parameters	Δρ_max_ = 1.12 e Å^−^^3^
10 restraints	Δρ_min_ = −0.56 e Å^−^^3^

### Special details

**Table:**

Geometry. All esds (except the esd in the dihedral angle between two l.s. planes) are estimated using the full covariance matrix. The cell esds are taken into account individually in the estimation of esds in distances, angles and torsion angles; correlations between esds in cell parameters are only used when they are defined by crystal symmetry. An approximate (isotropic) treatment of cell esds is used for estimating esds involving l.s. planes.
Refinement. Refinement of F^2^ against ALL reflections. The weighted R-factor wR and goodness of fit S are based on F^2^, conventional R-factors R are based on F, with F set to zero for negative F^2^. The threshold expression of F^2^ > 2sigma(F^2^) is used only for calculating R-factors(gt) etc. and is not relevant to the choice of reflections for refinement. R-factors based on F^2^ are statistically about twice as large as those based on F, and R- factors based on ALL data will be even larger.

### Fractional atomic coordinates and isotropic or equivalent isotropic displacement parameters (Å^2^)

**Table:**

	*x*	*y*	*z*	*U*_iso_*/*U*_eq_	
Ni1	1.0000	0.0000	0.0000	0.0311 (3)	
O1	0.9986 (3)	0.1074 (2)	0.1014 (3)	0.0408 (7)	
O2	0.5354 (7)	0.2034 (9)	0.9557 (8)	0.225 (5)	
O3	0.4032 (8)	0.1988 (7)	0.8277 (7)	0.192 (4)	
O4	0.3971 (5)	0.2888 (5)	0.9549 (6)	0.136 (3)	
N1	0.8796 (3)	−0.0671 (3)	0.0330 (3)	0.0345 (8)	
N2	0.5785 (3)	0.0578 (3)	−0.2176 (4)	0.0545 (11)	
N3	0.4423 (5)	0.2296 (4)	0.9150 (5)	0.0721 (14)	
C1	0.9022 (4)	0.0217 (3)	0.2196 (4)	0.0352 (9)	
C2	0.9667 (4)	0.1028 (3)	0.1957 (4)	0.0359 (9)	
C3	0.9970 (4)	0.1886 (4)	0.2757 (4)	0.0441 (11)	
H3	1.0384	0.2466	0.2589	0.053*	
C4	0.9685 (4)	0.1902 (4)	0.3755 (4)	0.0473 (11)	
H4	0.9901	0.2484	0.4212	0.057*	
C5	0.9090 (4)	0.1069 (4)	0.4066 (4)	0.0421 (10)	
C6	0.8845 (4)	0.1068 (5)	0.5149 (4)	0.0547 (13)	
H6	0.9079	0.1643	0.5614	0.066*	
C7	0.8303 (5)	0.0261 (5)	0.5452 (5)	0.0591 (15)	
H7	0.8141	0.0244	0.6126	0.071*	
C8	0.7973 (4)	−0.0588 (5)	0.4694 (4)	0.0562 (14)	
H8	0.7590	−0.1169	0.4898	0.067*	
C9	0.8189 (4)	−0.0615 (4)	0.3639 (4)	0.0491 (12)	
H9	0.7943	−0.1197	0.3187	0.059*	
C10	0.8757 (4)	0.0217 (3)	0.3293 (4)	0.0394 (10)	
C11	0.8539 (4)	−0.0524 (3)	0.1298 (4)	0.0371 (10)	
H11	0.7939	−0.0985	0.1407	0.045*	
C12	0.8045 (4)	−0.1365 (3)	−0.0545 (4)	0.0413 (10)	
H12A	0.7442	−0.1746	−0.0276	0.050*	
H12B	0.8601	−0.1871	−0.0727	0.050*	
C13	0.7332 (4)	−0.0759 (4)	−0.1585 (4)	0.0456 (11)	
H13A	0.7917	−0.0425	−0.1911	0.055*	
H13B	0.6779	−0.1220	−0.2125	0.055*	
C14	0.6604 (4)	0.0017 (4)	−0.1200 (5)	0.0541 (13)	
H14A	0.7168	0.0506	−0.0706	0.065*	
H14B	0.6090	−0.0323	−0.0805	0.065*	
C15	0.4901 (5)	−0.0100 (5)	−0.2939 (8)	0.095 (3)	
H15A	0.5335	−0.0566	−0.3306	0.114*	
H15B	0.4280	0.0304	−0.3491	0.114*	
C16	0.4293 (7)	−0.0697 (6)	−0.2234 (11)	0.162 (5)	
H16A	0.4811	−0.1288	−0.1918	0.243*	
H16B	0.3476	−0.0937	−0.2673	0.243*	
H16C	0.4201	−0.0252	−0.1656	0.243*	
C17	0.6274 (5)	0.1202 (5)	−0.2952 (6)	0.083 (2)	
H17A	0.6633	0.0743	−0.3385	0.100*	
H17B	0.5594	0.1602	−0.3449	0.100*	
C18	0.7222 (6)	0.1889 (6)	−0.2297 (10)	0.135 (4)	
H18A	0.6979	0.2124	−0.1674	0.203*	
H18B	0.7311	0.2482	−0.2731	0.203*	
H18C	0.8014	0.1523	−0.2045	0.203*	
H2	0.548 (6)	0.100 (4)	−0.176 (4)	0.080*	

### Atomic displacement parameters (Å^2^)

**Table:**

	*U*^11^	*U*^22^	*U*^33^	*U*^12^	*U*^13^	*U*^23^
Ni1	0.0217 (4)	0.0292 (4)	0.0426 (5)	−0.0016 (3)	0.0100 (3)	0.0006 (3)
O1	0.0354 (15)	0.0351 (16)	0.056 (2)	−0.0065 (12)	0.0206 (14)	−0.0047 (14)
O2	0.122 (6)	0.287 (12)	0.234 (9)	0.107 (7)	0.006 (6)	−0.103 (9)
O3	0.162 (6)	0.188 (7)	0.191 (6)	0.051 (5)	−0.001 (5)	−0.087 (6)
O4	0.086 (4)	0.132 (5)	0.223 (7)	0.024 (4)	0.093 (4)	−0.033 (5)
N1	0.0218 (15)	0.0313 (18)	0.047 (2)	−0.0011 (12)	0.0060 (14)	−0.0014 (15)
N2	0.0246 (17)	0.055 (3)	0.080 (3)	0.0071 (17)	0.0115 (18)	−0.009 (2)
N3	0.046 (3)	0.081 (4)	0.096 (4)	0.009 (3)	0.032 (3)	−0.010 (3)
C1	0.0223 (17)	0.036 (2)	0.046 (2)	0.0032 (15)	0.0096 (16)	0.0034 (17)
C2	0.0235 (17)	0.038 (2)	0.046 (2)	0.0045 (15)	0.0097 (17)	0.0011 (18)
C3	0.035 (2)	0.041 (3)	0.056 (3)	−0.0073 (18)	0.013 (2)	−0.004 (2)
C4	0.033 (2)	0.052 (3)	0.053 (3)	0.0012 (19)	0.007 (2)	−0.014 (2)
C5	0.0236 (18)	0.054 (3)	0.048 (3)	0.0068 (18)	0.0093 (18)	0.000 (2)
C6	0.039 (2)	0.072 (4)	0.053 (3)	0.010 (2)	0.015 (2)	−0.007 (3)
C7	0.041 (3)	0.093 (4)	0.051 (3)	0.017 (3)	0.025 (2)	0.008 (3)
C8	0.030 (2)	0.078 (4)	0.065 (3)	0.006 (2)	0.020 (2)	0.021 (3)
C9	0.029 (2)	0.057 (3)	0.063 (3)	0.003 (2)	0.018 (2)	0.008 (2)
C10	0.0194 (17)	0.049 (3)	0.049 (3)	0.0084 (16)	0.0103 (17)	0.0050 (19)
C11	0.0235 (18)	0.035 (2)	0.052 (3)	0.0008 (16)	0.0106 (17)	0.0072 (19)
C12	0.0222 (18)	0.038 (2)	0.062 (3)	−0.0043 (16)	0.0096 (18)	−0.011 (2)
C13	0.0254 (19)	0.049 (3)	0.058 (3)	0.0024 (18)	0.0058 (19)	−0.011 (2)
C14	0.030 (2)	0.067 (3)	0.065 (3)	0.014 (2)	0.013 (2)	−0.004 (3)
C15	0.031 (3)	0.070 (4)	0.156 (8)	0.007 (3)	−0.015 (4)	−0.016 (4)
C16	0.043 (4)	0.079 (5)	0.338 (16)	−0.010 (4)	0.019 (6)	0.054 (8)
C17	0.045 (3)	0.081 (4)	0.126 (6)	0.018 (3)	0.029 (3)	0.032 (4)
C18	0.044 (4)	0.083 (5)	0.270 (13)	−0.003 (3)	0.035 (5)	0.015 (7)

### Geometric parameters (Å, °)

**Table:**

Ni1—N1^i^	1.743 (3)	C7—H7	0.9300
Ni1—N1	1.743 (3)	C8—C9	1.437 (7)
Ni1—O1	1.888 (3)	C8—H8	0.9300
Ni1—O1^i^	1.888 (3)	C9—C10	1.374 (6)
O1—C2	1.353 (5)	C9—H9	0.9300
O2—N3	1.059 (7)	C11—H11	0.9300
O3—N3	1.135 (8)	C12—C13	1.531 (6)
O4—N3	1.111 (6)	C12—H12A	0.9700
N1—C11	1.360 (5)	C12—H12B	0.9700
N1—C12	1.469 (5)	C13—C14	1.454 (6)
N2—C15	1.442 (7)	C13—H13A	0.9700
N2—C14	1.483 (7)	C13—H13B	0.9700
N2—C17	1.495 (8)	C14—H14A	0.9700
N2—H2	0.89 (6)	C14—H14B	0.9700
C1—C2	1.345 (6)	C15—C16	1.487 (12)
C1—C11	1.457 (6)	C15—H15A	0.9700
C1—C10	1.513 (6)	C15—H15B	0.9700
C2—C3	1.465 (6)	C16—H16A	0.9600
C3—C4	1.400 (7)	C16—H16B	0.9600
C3—H3	0.9300	C16—H16C	0.9600
C4—C5	1.373 (7)	C17—C18	1.428 (10)
C4—H4	0.9300	C17—H17A	0.9700
C5—C10	1.439 (6)	C17—H17B	0.9700
C5—C6	1.484 (7)	C18—H18A	0.9600
C6—C7	1.310 (8)	C18—H18B	0.9600
C6—H6	0.9300	C18—H18C	0.9600
C7—C8	1.424 (8)		
			
N1^i^—Ni1—N1	180.0 (3)	C9—C10—C1	122.2 (4)
N1^i^—Ni1—O1	88.51 (14)	C5—C10—C1	123.4 (4)
N1—Ni1—O1	91.49 (14)	N1—C11—C1	132.3 (4)
N1^i^—Ni1—O1^i^	91.49 (14)	N1—C11—H11	113.8
N1—Ni1—O1^i^	88.51 (14)	C1—C11—H11	113.8
O1—Ni1—O1^i^	180.0 (2)	N1—C12—C13	112.1 (4)
C2—O1—Ni1	129.4 (3)	N1—C12—H12A	109.2
C11—N1—C12	123.0 (3)	C13—C12—H12A	109.2
C11—N1—Ni1	122.5 (3)	N1—C12—H12B	109.2
C12—N1—Ni1	114.4 (3)	C13—C12—H12B	109.2
C15—N2—C14	113.2 (5)	H12A—C12—H12B	107.9
C15—N2—C17	100.5 (6)	C14—C13—C12	104.3 (4)
C14—N2—C17	124.0 (4)	C14—C13—H13A	110.9
C15—N2—H2	117 (4)	C12—C13—H13A	110.9
C14—N2—H2	93 (4)	C14—C13—H13B	110.9
C17—N2—H2	110 (4)	C12—C13—H13B	110.9
O2—N3—O4	120.0 (8)	H13A—C13—H13B	108.9
O2—N3—O3	113.3 (7)	C13—C14—N2	108.0 (4)
O4—N3—O3	126.3 (7)	C13—C14—H14A	110.1
C2—C1—C11	114.7 (4)	N2—C14—H14A	110.1
C2—C1—C10	118.4 (4)	C13—C14—H14B	110.1
C11—C1—C10	126.7 (4)	N2—C14—H14B	110.1
C1—C2—O1	122.3 (4)	H14A—C14—H14B	108.4
C1—C2—C3	116.4 (4)	N2—C15—C16	103.9 (7)
O1—C2—C3	121.3 (4)	N2—C15—H15A	111.0
C4—C3—C2	125.2 (4)	C16—C15—H15A	111.0
C4—C3—H3	117.4	N2—C15—H15B	111.0
C2—C3—H3	117.4	C16—C15—H15B	111.0
C5—C4—C3	120.8 (4)	H15A—C15—H15B	109.0
C5—C4—H4	119.6	C15—C16—H16A	109.5
C3—C4—H4	119.6	C15—C16—H16B	109.5
C4—C5—C10	115.7 (4)	H16A—C16—H16B	109.5
C4—C5—C6	120.6 (5)	C15—C16—H16C	109.5
C10—C5—C6	123.7 (4)	H16A—C16—H16C	109.5
C7—C6—C5	120.3 (5)	H16B—C16—H16C	109.5
C7—C6—H6	119.8	C18—C17—N2	107.0 (7)
C5—C6—H6	119.8	C18—C17—H17A	110.3
C6—C7—C8	116.5 (5)	N2—C17—H17A	110.3
C6—C7—H7	121.8	C18—C17—H17B	110.3
C8—C7—H7	121.8	N2—C17—H17B	110.3
C7—C8—C9	124.8 (5)	H17A—C17—H17B	108.6
C7—C8—H8	117.6	C17—C18—H18A	109.5
C9—C8—H8	117.6	C17—C18—H18B	109.5
C10—C9—C8	120.4 (5)	H18A—C18—H18B	109.5
C10—C9—H9	119.8	C17—C18—H18C	109.5
C8—C9—H9	119.8	H18A—C18—H18C	109.5
C9—C10—C5	114.3 (4)	H18B—C18—H18C	109.5

Symmetry codes: (i) −*x*+2, −*y*, −*z*.

### Hydrogen-bond geometry (Å, °)

**Table:**

*D*—H···*A*	*D*—H	H···*A*	*D*···*A*	*D*—H···*A*
N2—H2···O3^ii^	0.89 (6)	2.05 (4)	2.836 (8)	146 (6)
N2—H2···O2^ii^	0.89 (6)	2.17 (5)	3.033 (9)	162 (6)

Symmetry codes: (ii) *x*, *y*, *z*−1.
